# Dendritic Spines in Alzheimer’s Disease: How the Actin Cytoskeleton Contributes to Synaptic Failure

**DOI:** 10.3390/ijms21030908

**Published:** 2020-01-30

**Authors:** Silvia Pelucchi, Ramona Stringhi, Elena Marcello

**Affiliations:** Department of Pharmacological and Biomolecular Sciences, Università degli Studi di Milano, 20133 Milan, Italy; silvia.pelucchi@unimi.it (S.P.); stringhi.ramona@gmail.com (R.S.)

**Keywords:** synaptopathy, actin cytoskeleton, actin-binding proteins, amyloid, synaptic plasticity

## Abstract

Alzheimer’s disease (AD) is a neurodegenerative disorder characterized by Aβ-driven synaptic dysfunction in the early phases of pathogenesis. In the synaptic context, the actin cytoskeleton is a crucial element to maintain the dendritic spine architecture and to orchestrate the spine’s morphology remodeling driven by synaptic activity. Indeed, spine shape and synaptic strength are strictly correlated and precisely governed during plasticity phenomena in order to convert short-term alterations of synaptic strength into long-lasting changes that are embedded in stable structural modification. These functional and structural modifications are considered the biological basis of learning and memory processes. In this review we discussed the existing evidence regarding the role of the spine actin cytoskeleton in AD synaptic failure. We revised the physiological function of the actin cytoskeleton in the spine shaping and the contribution of actin dynamics in the endocytosis mechanism. The internalization process is implicated in different aspects of AD since it controls both glutamate receptor membrane levels and amyloid generation. The detailed understanding of the mechanisms controlling the actin cytoskeleton in a unique biological context as the dendritic spine could pave the way to the development of innovative synapse-tailored therapeutic interventions and to the identification of novel biomarkers to monitor synaptic loss in AD.

## 1. Introduction

Alzheimer’s disease (AD) is the most common cause of dementia, characterized by decline in memory and thinking and by the impairment of at least two domains of cognition [1]. AD progression is associated with a significant and progressive disability throughout the disease course, with death generally occurring within 5–12 years of symptom onset [2]. Therefore, the burden on caregivers and the public health sector is enormous, leading to high nonmedical cost [3]. Unfortunately, therapies that may prevent or slow the rate of AD progression are not available. In such a scenario, it is fundamental to develop a disease-modifying intervention to produce an enduring change in the clinical progression of AD by interfering with the pathophysiological mechanisms.

**Figure 1:**
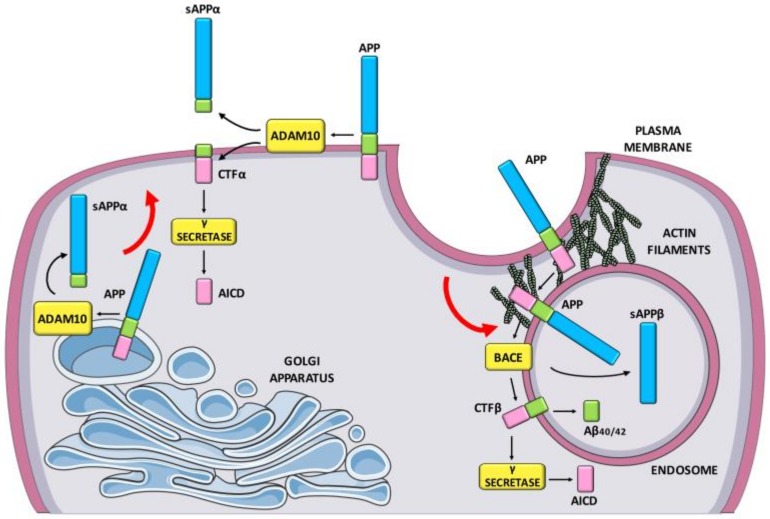
Schematic representation of β-amyloid precursor protein (APP) processing. The vast majority of BACE1 cleavage of APP occurs into BACE1-containing acidic organelles via clathrin-dependent endocytosis. The subsequent γ-secretase activity determines the release of Aβ. Alternatively, APP can undergo a non-amyloidogenic pathway that involves the α-secretase ADAM10 and occurs largely on the plasma membrane and in the trans-Golgi network. Red arrow, exocytosis/endocytosis pathways; black arrows, enzymatic cleavage of APP.

So far, AD pathogenesis relies on the amyloid hypothesis [4], according to which the Amyloid-β (Aβ) peptide aggregation plays a critical role at the beginning of the cascade of events leading to dementia. Aβ is a small peptide of 40–42 amino acids that was identified as the main constituent of amyloid neuritic plaques [5]. The concerted action of β-secretase BACE1 and γ-secretase determines the release of Aβ from the β-amyloid precursor protein (APP) [6]. Neuronal activity drives APP into BACE1-containing acidic organelles via clathrin-dependent endocytosis [7], where the vast majority of BACE1 cleavage of APP occurs [8] (Figure 1). Alternatively, APP can undergo a non-amyloidogenic pathway that involves the α-secretase ADAM10, a metalloprotease able to cleave APP within the sequence corresponding to Aβ [9,10]. ADAM10 cleavage not only prevents Aβ generation but also increases the release of the neurotrophic and neuroprotective sAPPα fragment [11]. The non-amyloidogenic ADAM10 processing of APP occurs largely on the plasma membrane [12] and in the trans-Golgi network [13] (Figure 1). APP and the secretases are all transmembrane proteins. Therefore, the trafficking mechanisms can control APP shedding and Aβ generation in neuronal cells. For example, ADAM10 synaptic localization and activity towards APP are finely tuned by its binding partners SAP97 and the clathrin adaptor protein AP2, that regulate ADAM10 forward trafficking and endocytosis respectively [14,15,16,17]. Moreover, perturbation of BACE1 post-Golgi trafficking results in an increase in BACE1 cleavage of APP and increased production of Aβ [18].

In addition to amyloid plaques, intracellular deposits of hyperphosphorylated tau protein, named neurofibrillary tangles, represent the other main hallmark for AD. Several evidences suggest that the increase in Aβ levels may trigger the progression of tau pathology in AD. For instance, experiments performed in a three-dimensional in vitro human neural cell culture system revealed that elevated Aβ levels alone are sufficient to drive tau pathology in human neurons [19]. Furthermore, the injection of Aβ fibrils into tau transgenic mice and the generation of animal models harboring both pathologies have demonstrated that Aβ accelerates tau pathology and neurodegeneration, whereas Aβ pathology is generally unaffected by concurrent tau pathology [20,21,22,23,24].

However, Aβ leads to cognitive impairment independent of its effects on tau pathology. Soluble Aβ oligomers isolated from the AD brain have been shown to induce synaptic loss [25] and to deeply affect the activity-dependent synaptic plasticity phenomena, such as long-term potentiation (LTP) and long-term depression (LTD) [26]. Moreover, Aβ oligomers cause impairment in cognitive tasks when injected into the lateral ventricles of rodent models [27]. Plasticity is a normal and essential part of cognition and LTP and LTD are considered the biological basis of learning and memory formation processes. Therefore, the small Aβ oligomers impair the main functional units of the brain and negatively affect their capability to store memories. These results have prompted the idea that AD is principally a disorder of synaptic function, i.e., a “synaptopathy” [28]. Indeed, late stage AD involves an incontrovertible and substantial loss of neurons and synapses [29]. Moreover, data obtained in the brains of AD patients and of subjects affected by Mild Cognitive Impairment showed that synapse loss is an early event in the disease process and a structural correlate involved in cognitive decline [30], thus supporting the idea of AD as synaptopathy.

Considering that Aβ oligomer-triggered synaptic dysfunction is causally linked to the early cognitive symptoms detected in AD patients, it is fundamental to decipher which synaptic pathways are affected in early phases of AD. Here we review the role of the actin cytoskeleton in dendritic spines as one of the main biological pathways crucial for both synaptic plasticity and synaptic pathology in early stages of AD. The identification of the synaptic molecules involved in the Aβ-mediated actin cytoskeleton failure in spines will be fundamental to developing synapse-tailored therapies and to assess in vivo biomarkers able to track synaptic integrity over time in patients.

## 2. The Actin Cytoskeleton as the Architect of Spines

In the mammalian brain, the postsynaptic compartment of glutamatergic excitatory synapses is localized in small protrusions along dendrites named dendritic spines [31,32]. Mature spines are characterized by a mushroom shape consisting of a head connected to the dendrite shaft by a narrower neck, while “stubby” spines lack the neck and filopodia-like are “headless” spines [31]. This distinctive morphology depends on an underlying cytoskeletal structure [33]. Whereas the dendritic shaft cytoplasm is dominated by microtubules, actin is the major cytoskeletal component of dendritic spines [34], where it is organized in a complex network of long and short branching filaments within the spine neck and in the spine head [35]. The actin cytoskeleton is a very dynamic structure able to self-assemble its building block G-actin (globular and monomeric form) in an ATP hydrolysis-dependent manner. This reaction generates a filamentous structure called F-actin (polymeric state) that can disassemble back into the monomer pool. G-actin is arranged head-to-tail to give the filament a molecular polarity, so F-actin is an asymmetric polymer with two ends that are dynamically different, called barbed and pointed ends. The first one is the more dynamic end, in fact, it elongates 10 times faster than the pointed end [36]. These processes occur on short time scales, allowing the cell to rapidly respond to internal or external stimuli [37,38]. Indeed, both the monomeric and filamentous forms of actin are present in the spine and the G-actin/F-actin ratio influences the various aspects of dendritic spine morphology and synaptic function [39]. 

The spine cytoskeleton is localized in the region just underneath the postsynaptic density (PSD) and is closely associated with a disk-shaped array of proteins attached to the postsynaptic membrane. The PSD provides a structural framework for postsynaptic signaling and plasticity [40]. Indeed, the PSD is fundamental for localizing molecules, including glutamate receptors and signaling molecules, in a functional organized structure of scaffolding proteins [40]. Importantly, scaffolding proteins of the PSD, such as SHANK and PSD-95, are associated with the dendritic actin cytoskeleton through interaction with actin F-binding proteins like cortactin and α-actinin [41,42]. In particular, dendritic spines contain two different pools of F-actin: (i) a very dynamic pool exists below the spine surface and interacts directly or indirectly with AMPA receptors, NMDA receptors, and PSD scaffolding and signaling proteins; (ii) a more internal and stable pool of F-actin serves as the main scaffold that supports the overall spine structure [35,43]. More recently, super-resolution microscopy techniques revealed periodic actin structures in dendrites and in the neck of dendritic spines [44,45] that may provide mechanical support and elasticity to this structure [46].

### 2.1. Synaptic Actin-Binding Proteins Orchestrating Actin Cytoskeleton Dynamics

The general function of actin is to allow the cell to respond to internal or external stimuli and, therefore, its capability to self-assemble is specifically spatiotemporally controlled [37,38]. To modulate the complex dynamic of the actin filaments there are several different proteins, called actin-binding proteins, able to bind and regulate actin dynamics. Cooperatively, the actin-binding proteins maintain a large pool of G-actin monomers available for polymerization, nucleate assembly of new filaments, promote elongation, cap barbed or pointed ends to terminate elongation, sever filaments, and cross-link filaments (reviewed in [47]). Therefore, the actin-binding proteins regulate the physiology of actin, giving to the actin the characteristic dynamism and stability.

In dendritic spines the most important and investigated actin-binding proteins are:

#### 2.1.1. Actin-Related Proteins-2/3

Actin-Related Proteins-2/3 (Arp2/3) are a complex made of different subunits, among which are Arp2, Arp3, ARPC1, ARPC2, ARPC3, ARPC4, and ARPC. Arp2/3 is the principal actin filaments nucleator [35] and, thereby, the major actin-binding protein with polymerizing and filament branching activities [48]. It can bind the filamentous actin to both sides and allows the insertion and creation of an additional filament [49]. It is enriched in the PSD and its downregulation results in an impairment in the spine head formation [50,51]. Several proteins, such as Cortactin, Abi2, WAVE-1 (WASp-family verprolin homology protein-1), N-WASP (neural Wiskott-Aldrich syndrome protein), and Abp1 activate Arp2/3 and their deletion is associated to memory deficits [52,53,54,55,56].

#### 2.1.2. Profilin

This class of proteins is fundamental for actin polymerization, since they are responsible for the ADP to ATP nucleotide exchange on actin. These proteins catalyze actin polymerization in a concentration-dependent manner: they are catalysts at lower concentrations and inhibitors at higher levels [57]. Profilin 2 is the principal isoform in the mammalian brain [35], even if profilin 1 is also expressed [58].

#### 2.1.3. Rho Family of GTPases

There are different components of this family of Ras proteins, including Ras homolog gene family member A (RhoA), Ras-related C3 botulinum toxin substrate 1 (Rac1), and Cell division control protein 42 homolog (Cdc42). All the members of this family have been studied in neuronal cells since they are involved in the neuronal morphogenesis. In the dendritic spine, the Rho activation is fundamental for the cofilin phosphorylation and therefore for the actin stabilization of the spine [35]. On the other hand, Rac1 and Cdc42 activation lead to an enlargement of the head spine [51], promoting the formation of Arp2/3 complex.

#### 2.1.4. ADF/Cofilin

The cofilin isoforms, i.e., cofilin-1 and cofilin-2, belong to a highly conserved protein family, as the actin depolymerizing factor (ADF) [59]. Cofilin can promote the actin turnover because it exerts a bidirectional effect on F-actin, depending on its relative concentration to actin. At low concentrations, cofilin promotes F-actin disassembly by cutting the actin filaments (severing) or by facilitating the removal of the actin monomers (depolymerization) [60]. F-actin severing can actually result in an increased rate of actin polymerization if there are enough actin monomers available, due to the creation of free barbed filament ends. The role of this complex is fundamental for continuous treadmilling of actin. In fact, since the actin monomers are fundamental for a fast reorganization of the actin cytoskeleton, the complex ADF/cofilin promotes the depolymerization of actin and creates a new pool of G-actin monomers available for the formation of other filaments [61]. The result of this activity is the correct maintenance for the morphology of the spine [51]. Cofilin is inactivated by phosphorylation on Ser3 by LIM kinase 1 (LIMK1) [62] and activated by slingshot homolog 1 (SSH1)-mediated dephosphorylation of Ser3 [63]. The actin interacting protein 1 (Aip1) is a cofilin accessory protein that selectively binds to cofilin-decorated actin filaments to induce the capping [64,65] or the destabilization of the filaments [66].

#### 2.1.5. Cyclase-Associated Proteins

Cyclase-associated proteins (CAP) can control filament turnover by recycling actin monomers and severing actin filaments [67]. In particular, it has been shown that the N-terminal region of the yeast homolog Svr2/CAP is responsible for the role that CAP plays in synergy with cofilin to accelerate actin filament depolymerization [68,69,70,71]. In mammals, two CAP homologs are expressed: CAP1 shows a wide tissue distribution, whereas CAP2 is primarily present in brain, heart, and skeletal muscle, skin, and testis [72], suggesting that these proteins have distinct functional roles. The deletion of CAP2 affects the F-actin/G-actin ratio in neurons, leading to an accumulation of F-actin in intracellular structures. The lack of CAP2 modifies neuronal architecture and spine morphology. CAP2 C-terminal domain interacts with cofilin, and such association depends on cofilin phosphorylation on Ser3. The ablation of CAP2 leads to increased levels of dephosphorylated (active) cofilin along with cofilin intracellular aggregation [73].

#### 2.1.6. Epidermal Growth Factor Receptor Pathway Substrate 8

Epidermal Growth Factor Receptor Pathway Substrate 8 (Eps8) is a capping protein. Capping proteins are involved in actin polymerization through the bond with barbed ends of F-actin, in order to block addition and removal of actin subunits [74]. The capping proteins are distributed in the dendritic spine and their function is relevant to inhibiting the filopodia formation [75]. The actin-capping proteins control the organization of filopodia. They can cap the newly branched filaments created by the Arp2/3 complex, thus controlling the elongation of the filopodia and the concentration of the free G-actin monomers.

#### 2.1.7. Myosins V and VI

Myosins V and VI are actin-based motor proteins that hydrolyze ATP to generate the mechanical force required for movement along actin filaments [76,77]. This enables myosins to propel the sliding of actin filaments, to produce tension on actin filaments, and to walk along these filaments. As a result, myosins can regulate the structure and dynamics of the actin cytoskeleton and affect the localization and transport of cellular components [76]. Motor proteins can be classified in either plus- or minus-end-directed motors. Plus-directed motor proteins move towards the barbed (plus) end of actin filaments, directing the cargo to the cell periphery, while minus-end-directed motors move towards the pointed (minus) end of actin filaments and have a major role in the movement of endocytic vesicles away from the plasma membrane [78]. The myosins V and VI are involved in the forward trafficking and internalization of the AMPA receptor [76].

#### 2.1.8. Tropomyosins

Tropomyosins (Tpms) are a family of actin-associated proteins relevant for the regulation of the actin cytoskeleton. Tpms are organized in coiled-coil dimers that form a head-to-tail polymer along the length of actin filaments. In mammals there four Tpm genes that can produce a range of Tpm isoforms by alternative exon splicing [79], but in neuronal cells isoforms deriving from *TPM1*, *TPM3*, and *TPM4* genes are found [80]. Tpms control the interaction of actin filaments with myosin motors and actin-binding proteins in an isoform-specific manner [81]. In particular, it has been shown that Tpm isoforms (i) bind along the sides of filaments and protect them from severing proteins and pointed-end depolymerization in vitro, (ii) affect the actin filament branching and nucleation, influencing the Arp2/3 complex activity [82], (iii) compete with ADF/cofilin family for actin binding in a Tpm isoform-dependent manner [83]. Neuronal-specific Tpm isoforms are differentially expressed, both temporally and spatially. Some Tpm isoforms have higher expression; during development their expression is required for the maintenance of the neuronal phenotype [84,85,86], whereas the expression of other isoforms increases with maturity [86,87]. For example, the Tpm3.1 isoform promotes axon length, cone size growth, and dendritic branching [88]. Such spatial and temporally controlled Tpm expression throughout the mouse brain, as well as in different sub-cellular compartments of neurons, is relevant for actin filament identity and specificity [85]. Indeed, while Tpm1.12 is found in the presynaptic compartment of cultured hippocampal neurons, *TPM3* and *TPM4* gene products localize to the postsynaptic region in mouse hippocampal neurons [89].

#### 2.1.9. Drebrin

Drebrin has two isoforms, embryonic-type drebrin E and adult-type drebrin A, that change during development from E to A [90]. Drebrin accumulates in dendritic spines where it creates a stable pool of slow turn-over F-actin and bundles filaments by crosslinking them together [91,92,93]. Drebrin associates with other actin-binding proteins such as myosins (I, II, V) and gelsolin [91,93] and provides a direct interaction with microtubules, since it associates to end-binding proteins that are microtubule plus-end tracking proteins localized to the growing plus-ends of dynamic microtubules [94]. Drebrin localization is regulated by activity-dependent synaptic plasticity and NMDA receptor activation [90].

#### 2.1.10. Ca^2+^/calmodulin dependent protein kinase II β

The Ca^2+^/calmodulin dependent protein kinase II (CaMKII) is a ubiquitous serine/threonine protein kinase that plays a key role in postsynaptic LTP and learning/memory [95,96]. Among the four CaMKII isoforms, the α and β subunits are the most abundant in the brain [97]. The β subunit features an F-actin-binding domain that targets CaMKIIβ to actin filaments in cells and particularly in dendritic spines [98]. CaMKIIα isoform weakly binds actin and this interaction is important for its localization to the dendritic spine [99]. On the other hand, CaMKIIβ does not only bind to actin, but also bundles actin filaments together [98] through the formation of hetero-oligomers with the CaMKIIα subunit [100]. Therefore, CaMKIIβ is a protein relevant for the maintenance of the spine structure and for plasticity-driven remodeling. CaMKIIβ appears to be anchored to a protein complex composed of drebrin-binding F-actin during the resting state. NMDA receptor activation releases CaMKIIβ from drebrin, resulting in CaMKIIβ association with PSD [101].

#### 2.1.11. α-actinin

α-actinin assembles in an antiparallel fashion and such dimers crosslink actin filaments [102]. The isoform α-actinin2 localizes to dendritic spines, enriched within the PSD and implicated in actin organization [103]. α-actinin selectively stabilizes CaMKII association with GluN2B-containing glutamate receptors [104]. The isoform α-actinin-4 is an interacting partner of metabotropic glutamate receptors and orchestrates spine dynamics and morphogenesis in neurons through a CaMKIIβ-dependent process [105].

### 2.2. The Actin Cytoskeleton in Spines: A Key Player of Activity-Dependent Synaptic Plasticity Events

During development and in adulthood, synapse formation, maintenance, and elimination come along with changes in dendritic spine number and morphology to establish and shape the connectivity of neuronal circuits [106]. The actin cytoskeleton is important for the stabilization of postsynaptic proteins in mature spines [107] and modulation of spine morphological adaptation (shape and number) in response to postsynaptic stimuli [108,109]. Indeed, at the cellular level, a synaptic function and spine shape modifications are strictly interdependent, especially during activity-dependent synaptic plasticity events, such as LTP and LTD [110]. In this framework the actin cytoskeleton dynamics are finely tuned since they are a critical element for the remodeling of the spine morphology, as well as for the endocytosis processes that control glutamate receptors levels at the membrane.

#### 2.2.1. Actin Cytoskeleton Remodeling to Change Spines Structure

**Figure 2:**
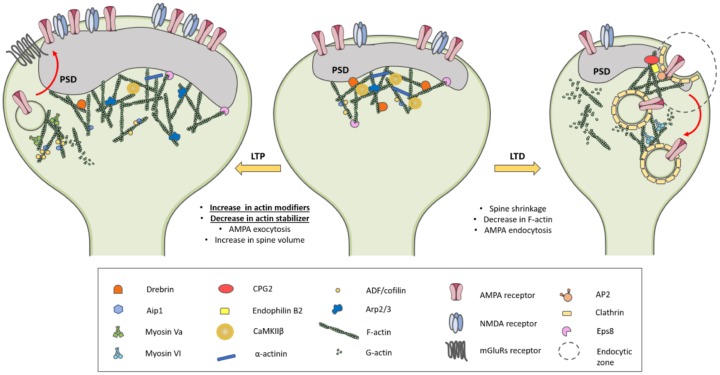
Dendritic spine actin cytoskeleton dynamics in activity-dependent synaptic plasticity phenomena. During the first phase of long-term potentiation (LTP) (1–7 min), there is a rearrangement of the actin cytoskeleton characterized by (i) the increase in the synaptic level of actin-binding proteins that are able to modify F-actin through several process, such as severing (cofilin), branching (Arp2/3), or capping (Aip1); (ii) the decrease in actin-stabilizer proteins (drebrin, CaMKIIβ, and α-actinin) responsible for stabilizing actin filaments and linking them to the postsynaptic density (PSD). After this phase, cofilin is inactivated to enlarge the spine and stabilize changes in structure. During long-term depression (LTD), the spine shrinkage is associated to a decrease in spine actin filaments. The actin cytoskeleton and actin-binding proteins are also involved in AMPA receptor trafficking: LTP triggers AMPA receptor forward trafficking, while LTD promotes clathrin-mediated endocytosis of AMPA receptors.

The equilibrium between F-actin and G-actin is stable under basal neuronal activity, but it can be rapidly modulated, both positively and negatively, by synaptic activity. Indeed, LTP shifts the G-actin/F-actin ratio toward F-actin (rise in spine actin filaments) and results in spine enlargement, while LTD shifts the G-actin/F-actin ratio toward G-actin (decrease in spine actin filaments) and results in spine shrinkage. Actin-binding proteins orchestrate these precise changes in actin cytoskeleton dynamics and have different localizations in spines during the different phases of LTP [109] (Figure 2).

The first phase of LTP is characterized by a transient but profound modification of the overall protein composition of the spine with a rapid increase of the actin levels and of actin polymerization in the spine [108]. During this phase of 1 to 7 min, the actin cytoskeleton is more unstable and susceptible to reorganization. In addition to the increase in F-actin, there is also a change in the composition of the actin-binding proteins in the spine. During these first minutes, the spine is significantly enriched in proteins able to largely modify F-actin through severing (cofilin), branching (Arp2/3), or capping (Aip1). At the same time, the concentration of proteins known to stabilize the suprastructure of the actin cytoskeleton by bundling F-actin or linking F-actin to the PSD (drebrin, CaMKIIβ, and α-actinin) is transiently reduced in the spine [109,111]. During this time window of about 5 min, therefore, actin filaments can lose their supramolecular organization (bundling and cross-linking) and allow the access to other actin-binding factors that can reorganize the actin cytoskeleton. This switch of actin-binding protein type from actin-stabilizers to actin-modifiers in the earliest phase of synapse potentiation creates a time window in which the actin cytoskeleton becomes susceptible to major reorganization (Figure 2). Afterwards, the concentration of drebrin, CaMKIIβ, and α-actinin in the spine progressively returns to basal levels of concentration [109].

One of the major players in such LTP-induced actin cytoskeleton remodeling is cofilin. It has been shown that upon the activation of the NMDA receptors, cofilin is translocated to the spine, where it severs the actin cytoskeleton, leading to the formation of new barbed ends that nucleate new filament growth [112]. The severing allows the formation of new F-actin, which is the preferred site of Arp2/3 nucleating and branching activity [48]. The concerted action of cofilin and Arp2/3 is fundamental for the maintenance of spine expansion and for the control of protein delivery to the synaptic membrane, such as AMPA receptors [47,113]. Indeed, the perturbation of cofilin inactivation by phosphorylation prevents the maintenance, but not the first phase, of spine enlargement, indicating that cofilin is required for the structural consolidation of the spine.

During the second phase (7–60 min after the LTP induction) the actin concentration goes back to basal levels, leading to the stabilization of the spine structure, while cofilin moves to the neck of the spine after its phosphorylation on Ser3 and, thereby, its inactivation. The complex cofilin-actin can stop the actin rearrangement, giving the spine the possibility to enlarge its structure. Indeed, after LTP induction, actin stabilizes the spine apparatus, representing an anchoring for several molecules [114]. In the third phase (corresponding to late LTP) that occurs 60 min after induction, the PSD is structurally remodeled by the increase in PSD proteins and newly synthesized factors [115]. Concerning the structure of the spine, it has been demonstrated that the F-actin nucleation, mediated by WAVE complex, an activator of the Arp2/3 complex, occurs in the central structure of the spine, while the elongation occurs at the tip of finger-like protrusions. For that reason, the proteins involved in the branching of the already assembled filaments are localized in the central part of the PSD, while next to the membrane the filament elongator proteins are confined. The synaptic plasticity modifies the distribution patterns of actin-binding proteins and induces also the redistribution of branched F-actin regulators in spines to create an enlargement also in the distal part of the spine [114].

#### 2.2.2. Actin Cytoskeleton and Endocytosis

Postsynaptic composition is tightly controlled and rapidly modulated during activity-dependent synaptic plasticity events. This synaptic property requires coordinated mechanisms of protein trafficking that are themselves under the control of the actin cytoskeleton.

For instance, the precise regulation of glutamate receptor number and subtype at the synapse, which is crucial to excitatory neurotransmission and synaptic plasticity, can be affected by actin cytoskeleton dynamics modulation. Cultured neurons exposed to latrunculin, a G-actin sequestering drug that blocks polymerization, showed reduced clustering of synaptic NMDA receptors and GluA1-containing AMPA receptors in dendritic spines [116] and specifically decreased AMPA receptor neurotransmission [117]. In addition, the F-actin stabilizing drug Jasplakinolide blocked glutamate-stimulated AMPAR internalization [118]. Indeed, AMPA receptors dynamically cycle between the plasma membrane and intracellular compartments through endo-exocytic events (reviewed in [119]). In addition to endocytosis and exocytosis, lateral diffusion of receptors in the plane of the membrane and exchange between synaptic and extrasynaptic sites emerged as key steps for modifying receptor numbers at synapses (reviewed in [120]). Constitutive endocytosis of AMPA receptors at the postsynaptic membrane is believed to be clathrin-independent [121], even though constitutive clathrin-mediated endocytosis (CME) of the receptor, as well as other cargos, was reported to occur in this subcompartment as well as in dendrites and in the soma [122]. On the other hand, it is widely accepted that the implementation of LTD requires CME of postsynaptic AMPA receptors [122,123,124] and is relevant for learning in vivo [125].

The CME is a complex process that requires coordination of the molecular events responsible for cargo sorting, membrane invagination, vesicle scission, and vesicle targeting. A vast body of research has revealed an intricate network of numerous protein-protein interactions within the endocytic pathway [126]. Genetic studies in yeast have firmly established a functional connection between actin and endocytosis, and experiments performed with drugs that interfere with actin cytoskeleton dynamics provided significant evidence that, in mammalian cells, CME relies on an active actin cytoskeleton [126]. For instance, the inhibition of actin polymerization by using latrunculin blocked clathrin-coated structure dynamics in neuronal dendrites [127].

In mammalian cells the CME was divided into several distinct stages that included coat assembly on membranes, invagination, fission, movement of vesicles away from the plasma membrane, and finally, uncoating [128,129]. Actin is crucial during these different stages of endocytic internalization and actin networks that form at sites of endocytosis must be tightly regulated for efficient internalization. Indeed, sites of endocytosis contain Arp2/3 to form a branched actin network, capping proteins, which limits the length of filaments and depolymerization factors such as cofilin, which turn over older filaments for recycling of actin subunits [130]. During the first phase, when endocytic coat proteins are recruited and the endocytic structure remains at the cell membrane undergoing relatively minor movements, the regulators of actin assembly are recruited, and near the end of this stage, actin polymerization begins. Therefore, actin is involved in specifying sites of coated-pit formation on the plasma membrane and may provide a scaffold for the assembly and anchoring of the endocytic machinery during clathrin-coated vesicle formation. It was shown that the clathrin-adaptor complex AP2, which mediates attachment of clathrin to the plasma membrane and induces clathrin polymerization into a coat, colocalizes with actin stress fibers [131]. The Huntingtin interacting protein 1 (HIP1) and Hip1R were suggested as potential linkers between the clathrin-coated pit and actin cytoskeleton. HIP1 binds AP2 and clathrin and is present in clathrin-coated vesicles [132], while Hip1R associates to both actin and clathrin [131]. The heterodimerization of HIP1 and Hip1R may be a mechanism that allows HIP1 to indirectly bind to F-actin and that also allows AP2 to be recruited to clathrin nucleation sites defined by the HIP proteins at the plasma membrane. During the second phase, the proteins make a short movement away from the membrane into the cytoplasm. At the end of this stage, endocytic proteins are lost from the vesicle, presumably coinciding with scission of the vesicle from the plasma membrane. At this stage, actin polymerization occurs at endocytic sites, thus providing a force during coated-pit neck constriction, fission, and detachment of clathrin-coated vesicles [133,134]. Using alternating evanescent field and epifluorescence illumination, Merrifield and colleagues showed that clathrin-coated pit invagination and scission are tightly coupled, with scission coinciding with maximal displacement of the clathrin-coated pit from the plasma membrane and with peak recruitment of cortactin, a dynamin and F-actin-binding protein. Indeed, perturbing actin polymerization reduces the efficiency of membrane scission [135]. In addition, a role for Tpm3.1 was hypothesized during the fission of the endosomes from the plasma membrane. Tpm3.1 can recruit non-muscle myosin II to stabilize actin filaments [136]. In light of this consideration, Tpm3.1 could be involved in maintaining the nascent endocytic neck and in stabilizing and recruiting myosin II, allowing the stabilization and constriction of the actin ring surrounding the neck of nascent bulk endosomes in preparation for their fission from the plasma membrane [137].

In dendritic spines, there are endocytosis-specialized regions named endocytic zones near the postsynaptic membrane and lateral to the PSD, where they develop and persist independent of synaptic activity, akin to the PSD itself [127]. In such specialized regions there are actin-binding proteins relevant for glutamate receptor endocytosis, such as the Candidate Plasticity Gene 2 (CPG2) that colocalizes with clathrin at postsynaptic endocytic zones [138,139]. CPG2, through a direct physical interaction, recruits endophilin B2 to F-actin, thus anchoring the endocytic machinery to the spine cytoskeleton and facilitating glutamate receptor internalization [124]. Specific disruption of endophilin B2 or the CPG2-endophilin B2 interaction impairs activity-dependent, but not constitutive, internalization of both NMDA- and AMPA-type glutamate receptors [124] (Figure 2).

As far as concern glutamate AMPA receptors, different linker proteins mediate the association of the receptor subunits to the actin cytoskeleton [113] and modulate AMPA receptor localization, internalization, and forward trafficking. The F-actin-binding proteins 4.1 were shown to stabilize AMPA receptors, providing a link to actin filaments [140], and to be involved in receptor exocytosis [141]. The deletion of the CAP2 protein impairs the LTP-triggered increase in GluA1 surface expression [73]. The protein PICK1, which is involved in LTD-induced internalization of AMPA receptors [142], binds GluA2/3 subunits, F-actin, and the Arp2/3 complex [143]. PICK1 is a negative regulator of Arp2/3-mediated actin polymerization that is critical for a specific form of vesicle trafficking and in particular for NMDA-induced AMPA internalization [143]. The reversion-induced LIM protein (RIL) is another protein linker that associates AMPA receptors with the actin cytoskeleton because it binds both the GluA1 C-terminus and the F-actin cross-linking protein α-actinin. It was proposed that such association plays a role in enhancing surface and synaptic expression of AMPA receptors by regulating endosomal recycling [144]. A protein relevant for AMPA receptor sorting is the actin-regulatory protein cortactin that interacts with the GluA2 subunit. Disrupting GluA2-cortactin binding in neurons causes the targeting of GluA2/A3-containing receptors to lysosomes and their consequent degradation, resulting in a loss of surface and synaptic GluA2 under basal conditions and an occlusion of subsequent LTD expression [145]. In addition, AMPA receptor trafficking is also dependent on the interaction with different myosins isoforms. Myosin Va, which is a plus-end-directed motor, binds GluA1 tail and is required for the LTP-induced forward trafficking of the receptor [146]. On the other hand, myosin VI exists in a complex with the AMPA receptors, AP2 and SAP97 in the brain, suggesting that myosin VI could play a role in the clathrin-mediated endocytosis of AMPA receptors [147] (Figure 2).

Sorting mechanisms are also important for amyloid generation in neuronal cells. Trafficking events play key roles in the convergence of APP and BACE1, putting in close proximity the substrate and the cleaving enzyme, respectively. Considering that BACE1 is optimally active in an acidic environment, the endosomes can be contemplated as the biological locus where the amyloidogenic pathway initiates [148]. Most studies on APP and BACE1 trafficking were done in non-neuronal cells and showed that trans-Golgi network, plasma membrane, and endosomes are the main sorting stations for APP and BACE1 [149]. In cultured hippocampal neurons, APP and BACE1 interact in both endoplasmic reticulum/Golgi and endocytic compartments, particularly in recycling zones such as dendritic spines and presynaptic boutons [150]. Abnormal residence time of APP and BACE1 in endosomes can lead to increased cleavage of APP, and this might contribute to sporadic AD [151]. After the synthesis, APP is conveyed in Golgi-derived vesicles to the plasma membrane where it can be internalized via a clathrin-dependent mechanism [7,152]. After internalization a significant population of APP endocytosed from the plasma membrane enters LAMP-1-positive late endosomes/lysosomes [150], while a portion of internalized APP can be routed into BACE1-positive recycling endosomes [7] Upon endocytosis APP can also be transported back to the trans-Golgi network via a retromer-dependent pathway [153]. A key regulator of the APP retrieval pathway is sorting protein-related receptor with A-type repeats (sorLA) [154], a scaffold protein linking APP to the retromer complex [155]. Overexpression of sorLA in neurons causes redistribution of APP to the Golgi and decreased processing to Aβ, whereas ablation of sorLA expression in knockout mice results in increased levels of Aβ in the brain [154]. Remarkably, genome-wide association studies showed an association of sorLA with sporadic, late-onset AD [156], strengthening the relevance of endocytosis in AD pathogenesis.

## 3. Actin Cytoskeleton Pathology in AD

Given the large contribution of the cytoskeleton to the mechanisms involved in synaptic plasticity, an impairment in actin cytoskeleton dynamics can contribute to AD pathology and underlie the synaptic failure in AD [28]. Several actin-binding proteins are altered in AD brains and AD animal models (Table 1). For example, the expression of drebrin is decreased in the hippocampus of aged AD mice compared with age-matched wild-type and young adult AD mice. In AD mice the overexpression of drebrin ameliorates cognitive ability and attenuates pathological lesions [157]. Moreover, the potential contribution of drebrin in AD synaptic failure is strengthened by studies showing its decrease in the hippocampal dendritic spines of AD brain patients [158] and in cortical areas, including the frontal and temporal cortices [159], in relation to the cognitive impairment associated with normal aging [160].

The involvement of the Rho family of small GTPases (Rho, Rac, and Cdc42) in AD pathology was shown in different AD models. In neuronal cultures exposed to fibrillar Aβ peptide, an increased localization and activity of Rac1/Cdc42 Rho GTPases, promoted by Tiam1, were observed together with a consequent enhancement in actin polymerization [161]. Rac1 is increased in plasma samples of AD patients [162] and, in the hippocampus of AD mice, Rac1 was abnormally activated at the early stages of the pathology, even if the total protein levels decreased at full-blown pathology stage. These data suggest that, in an initial stage, Rac1 deregulation might represent a triggering co-factor due to the direct effect on Aβ and tau [162].

Evidence of actin involvement in AD comes from studies of the main neuropathological hallmarks of AD, i.e., neurofibrillary tangles and the senile plaque, and of the additional lesions that are detectable in AD brains, such as the Hirano bodies and the actin rods. For example, the screening of 1250 mutant Drosophila lines allowed the identification of 30 specific modifiers of tau-induced neurodegeneration, among which were several components of the actin cytoskeleton, such as Tpm [163] and myosin VI, which colocalize with tau protein accumulated in the neurons of AD patients and of tauopathy animal models [164].

Hirano bodies are bright eosinophilic intracytoplasmic inclusions encountered within the CA1 region of the hippocampus. These lesions were first identified by Asao Hirano and their frequency increases with age and in a number of neurodegenerative diseases including AD [165,166]. Immunocytochemistry and immunoelectron-microscopy studies revealed the diffuse presence of actin, in the F-state, and cytoskeletal proteins throughout the Hirano body [167]. In addition to APP and several microtubule-associated proteins, actin-associated proteins such as Tpm, vinculin, α-actinin, ADF, and cofilin are components of these intracellular inclusions [168]. The association between AD and Tpm dysregulation can be isoform-specific. For example, a proteomics study showed that *TPM1* and *TPM3* gene products are increased in the white matter of AD patients when compared with controls [169]. In addition, a proteomic analysis of hippocampal glycoproteins revealed an increase in *TPM3* gene products in AD brains [170]. The strong presence of actin-binding proteins in the Hirano bodies supports the hypothesis that these lesions are derived from an abnormal organization of the neuronal cytoskeleton. To strengthen this hypothesis, it has been shown that profilin, Arp/2/3, WASH, and de novo actin polymerization are required for model Hirano body formation in *Dictyostelium* [171].

In addition to Hirano bodies, ADF and cofilin were detected in another form of inclusion, named actin rods, that is prominent in hippocampal and cortical neurites of the post-mortem brains of AD patients, especially in neurites contacting amyloid deposits [172,173], and in AD mice models [174]. Actin rods are cytoplasmic rod-shaped bundles of filaments composed of ADF/cofilin-actin in a 1:1 complex. In addition to cofilin and actin, cytoplasmic rods isolated from either cell lines or cultured primary neurons contain other proteins, but only during late stages of rod maturation, indicating that these are not core components of the rods. Vesicles containing APP, BACE1, and presenilin-1, a component of the γ-secretase complex, accumulate at rods, suggesting that actin rod formation blocks the transport of APP and enzymes involved in its processing to Aβ [174]. Rod formation has a local effect since transport deficits of mitochondria and early endosomes, a decline in spine numbers, and glutamate receptor response occur within neurites that form spontaneous rods, while neurites from the same neuron without rods remain unaffected [175].

In order to investigate the primary cause of actin rod formation, several neurodegenerative stimuli were investigated, such as exposure to excitotoxic levels of glutamate, mitochondrial inhibitors, peroxide, Aβ peptides, and proinflammatory cytokines [172,174]. ATP depletion is a major trigger for cofilin-actin rod formation at a stoichiometric proportion of 1:1 [172]. A decrease in ATP shifts the balance of kinase/phosphatase activity toward the active (dephosphorylated) form of cofilin, the augment of the ADP-actin fraction of total actin, and the increase of reactive oxygen species (ROS) [172,176]. In such environments, cofilin is able to saturate regions of F-actin that are readily severed, producing small stable fragments [177]. In the presence of high amounts of ROS, the newly generated F-actin fragments lead to the formation of rods through the direct bundling of these fragments and/or intermolecular disulfide cross-linking of cofilin [172,176]. In summary, the formation of actin rods requires (i) cofilin in the activated (dephosphorylated) form, (ii) cofilin saturation of local regions of F-actin in the presence of abnormally high levels of ADP-actin, which preferentially binds to cofilin, (iii) formation of intermolecular disulfide linkages via oxidation of several key cysteine residues of cofilin [178]. The formation of rods actually may also have a protective function. Considering that in neurons actin dynamics utilize a significant amount of ATP [179], in a short period (approximately 60 min) immediately after initial rod formation cofilin sequestration transiently delays the decline in mitochondrial membrane potential and helps maintain ATP levels by reducing actin filament turnover [180].

Even though the active dephosphorylated form is the main component of actin rods [174], data about the activation state of cofilin in AD are conflicting. Increased cofilin phosphorylation/inactivation with age and AD pathology was reported both in vivo and in vitro [181], while a decrease in cofilin phosphorylation was described in the frontal lobe of younger patients [182]. In APP/PS1 mice (a mouse model of AD) an increased cofilin activation/dephosphorylation was observed [183,184]. The analysis of the PSD-enriched fraction of both APP/PS1 mice and AD brains revealed a significant increase in cofilin phosphorylation/inactivation in the postsynaptic compartment [185]. Another study showed that cofilin phosphorylation state depends on the stage of the pathology, since an increased activation (dephosphorylation) is detected in APP/PS1 mice brains at four months of age while cofilin is strongly phosphorylated (inactivated) at 10 months of age, corresponding to a full-blown pathology stage [181].

Considering the crucial involvement of cofilin in several biological pathways related to the actin cytoskeleton, the alteration of cofilin activation and its sequestration in the actin rods could affect different cellular functions [60]. First, an alteration of cofilin levels and activation profoundly affects the biological pathways implicated in learning and memory processes. Indeed, cofilin is required for LTP, as demonstrated by the generation of cofilin knockout mice, which showed a complete lack of LTP in CA1 neurons of hippocampal slices, aberrant spine morphology, and impaired associative learning [186]. Cofilin controls extrasynaptic excitatory AMPA receptor diffusion [186], which affects the probability of a surface-expressed receptor being incorporated into the synapse [187], and cofilin activation is required for the insertion of the AMPA receptor subunit GluA1 upon LTP induction [188]. Second, the lack of cofilin can affect cellular transport. Indeed, cofilin directly competes with tau for microtubule binding and can be implicated in in tauopathy and destabilization of tau-regulated microtubule dynamics [189]. Third, cofilin is relevant for the regulation of mitochondria morphology and function [190,191]. In oxidative stress conditions, cofilin cysteine residues are oxidized, thus promoting intramolecular disulfide bridging of cofilin [192]. In such a state, cofilin loses affinity for actin and translocates to mitochondria, where it induces swelling, a drop in mitochondrial membrane potential, and cytochrome c release by promoting the opening of the permeability transition pore [192,193,194]. Mitochondrial targeting of cofilin and subsequent cytochrome c release mediate neuronal apoptosis in response to Aβ oligomers [183,195]. Finally, cofilin is also implicated in APP processing and Aβ metabolism. The knockdown of cofilin in primary neurons significantly reduces Aβ production by increasing surface APP levels. Expression of active, but not inactive, cofilin reduces membrane APP levels by enhancing APP endocytosis. In APP/PS1 mice, genetic reduction of cofilin reduces Aβ deposition together with significantly increased microglial activation that has a greater ability to uptake and clear Aβ. These results indicate a significant role for cofilin in Aβ accumulation via different neuronal and microglial mechanisms [196].

## 4. Conclusions

Even though in the last 25 years the pharmaceutical industry has invested in anti-amyloid therapies and in numerous phase 3 clinical trials, no amyloid-targeting therapy has improved or limited the progression of cognitive impairment in symptomatic AD. These data suggest that while amyloid accumulation may be key in beginning the pathological process, other downstream events such as synaptic loss and tau accumulation may be the main drivers of neurodegeneration [197]. In addition, considering that synaptic failure is causally linked to the early cognitive symptoms detected in AD patients, AD can be considered a synaptopathy, such as several other neurological disorders.

Notably, growing evidence demonstrates that the actin cytoskeleton plays a key role in synaptic function and plasticity. Therefore, the disturbance of actin-binding proteins, which orchestrate actin cytoskeleton dynamics, could be a common principle in many synaptopathies [198,199]. Several studies analyzing AD post-mortem tissue, animal and cellular models suggest that AD pathology has a deleterious effect on the pathways governing the actin cytoskeleton.

The actin cytoskeleton is a critical element during activity-dependent synaptic plasticity phenomena, since it takes part in different aspects of the coordinated machinery that modulates synaptic transmission. First, actin filament remodeling shapes the dendritic spine in response to the received stimulus. Secondly, actin polymerization is the driving force of clathrin-mediated endocytosis and actin-binding proteins are involved vesicle sorting. Therefore, the endocytosis controls not only the membrane levels of glutamate receptors but also the colocalization of the amyloid cascade players, thus affecting the production of Aβ peptide.

In light of these considerations, the actin cytoskeleton can be positioned at the crossroad of pathways contributing to AD pathogenesis, i.e., the amyloid cascade and the synaptic dysfunction. However, a great effort is needed to investigate in detail the mechanisms controlling actin cytoskeleton machinery in a specific cellular compartment that is the dendritic spine. Indeed, several studies on actin have been performed in test tubes and in yeast, but the main challenge is to understand the actions of actin and actin-binding proteins in a proper and highly specialized cellular context, i.e., the dendritic spine. The knowledge of the physiological mechanisms controlling synaptic plasticity is fundamental to understand the biological basis of memory formation and, thereby, to shed light on the pathological modifications that drive cognitive impairment in AD.

## Figures and Tables

**Table 1:** Actin-binding proteins: physiological role and involvement in AD.

Protein	Physiological Function	Role in AD
Arp2/3	Actin filaments nucleator: major actin-binding protein with polymerizing and filament branching activities	Required for Hirano body formation in *Dictyostelium*
Profilin	Responsible for the ADP to ATP nucleotide exchange on actin	Required for model Hirano body formation in *Dictyostelium*
Rho family of GTPases	Intermediaries between external signals and internal actin organization	- Increased activity of Rac1/Cdc42 Rho GTPases upon fibrillar Aβ exposure - Rac1 is increased in plasma samples of AD patients and abnormally activated in AD mice at early stages
ADF/cofilin	Promote the actin turnover	- Identified in the Hirano bodies and main component of actin rods -Alterations of its activation in in vitro and in vivo AD models
Myosins V and VI	Actin-based motor proteins	Myosin VI colocalizes with tau protein accumulated in neurons of AD patients
Drebrin	Bundles actin filaments by crosslinking	Decreased in the hippocampal dendritic spines of AD patients and in the hippocampus of AD mice
α-actinin	Form dimers that crosslink actin filaments	Component of the Hirano bodies
Tropomyosins	Form head-to-tail polymers to stabilize the actin filament and recruit myosin	Component of neurofibrillary pathology and Hirano bodies
